# *FOXP1* and *TP63* involvement in the progression of myelodysplastic syndrome with 5q- and additional cytogenetic abnormalities

**DOI:** 10.1186/1471-2407-14-396

**Published:** 2014-06-03

**Authors:** Alberto L’Abbate, Crocifissa Lo Cunsolo, Ettore Macrì, Paolo Iuzzolino, Cristina Mecucci, Claudio Doglioni, Michelina Coco, Lucia Anna Muscarella, Simona Salati, Enrico Tagliafico, Carla Minoia, Giacoma De Tullio, Attilio Guarini, Nicoletta Testoni, Claudio Agostinelli, Clelia Tiziana Storlazzi

**Affiliations:** 1 Department of Biology, University of Bari, Via G.Amendola 165/A, Bari 70126, Italy; 2 UO Anatomia Patologica, Ospedale S. Martino, Belluno, Italy; 3 Hematology Unit, University of Perugia, Polo Unico S.M. Misericordia, Perugia, Italy; 4 Istituto Scientifico San Raffaele, Milan, Italy; 5 Laboratory of Oncology, IRCCS “Casa Sollievo della Sofferenza”, San Giovanni Rotondo, Italy; 6 Center for Genome Research, Department of Biomedical Sciences, University of Modena and Reggio Emilia, Modena, Italy; 7 Haematology Unit, Department of Medical and Experimental Oncology, IRCCS National Cancer Research Centre “Giovanni Paolo II”, Bari, Italy; 8 “Seràgnoli” Institute of Hematology, Bologna University School of Medicine, Bologna, Italy

**Keywords:** Double inversion, Myeloid leukemia, TP53, Gene activation, Chromosome 3

## Abstract

**Background:**

The progression of low-risk del(5q) myelodysplastic syndrome to acute myeloid leukemia is increased when associated with mutations of *TP53,* or with additional chromosomal abnormalities. However, to date the prognostic impact and molecular consequences of these rearrangements were poorly investigated. Single additional alterations to del(5q) by balanced chromosome rearrangements were rarely found in myelodysplasia. In particular, balanced alterations involving *TP63* and *FOXP1* genes were never reported in the literature.

**Case presentation:**

Here we report on a 79-year woman with an aggressive form of myelodysplastic syndrome with del(5q), no *TP53* mutation, and a novel complex rearrangement of chromosome 3 in bone marrow cells. Our results revealed that the *FOXP1* and *TP63* genes were both relocated along chromosome 3. Strikingly, immunohistochemistry analysis showed altered protein levels, disclosing that this rearrangement triggered the expression of *FOXP1* and *TP63* genes. FOXP1 was also found activated in other patients with myelodysplasia and acute myeloid leukemia, showing that it is an important, recurrent event.

**Conclusions:**

We document an apparent role of FOXP1 and TP63, up to now poorly documented, in the progression of MDS in our patient who is lacking mutations in the *TP53* tumor suppressor gene normally associated with poor outcome in myelodysplastic syndrome with 5q-. Finally, our results may suggest a possible broader role of FOXP1 in the pathogenesis and progression of myelodysplasia and acute myeloid leukemia.

## Background

In MDS, del(5q) was associated with a low-risk of leukemic evolution, unless it was accompanied by a mutation of *TP53*[1], or by a complex karyotype (presence of ≥ 1 additional chromosomal abnormalities) [2,3]. The most frequent single extra abnormalities to del(5q) were: del(12p), trisomy 21, trisomy 8, del(20q) [2], and the recently identified del(15)(q26.1), and del(3)(q26.1), respectively deleting the *CHD2,* and *THPO* genes [3]. Conversely, balanced chromosome rearrangements were rarely found as a single additional alteration to del (5q). Notably, the molecular consequences and prognostic impact of each single aberration, mostly if balanced, were poorly investigated to date [4].

**Figure 1:**
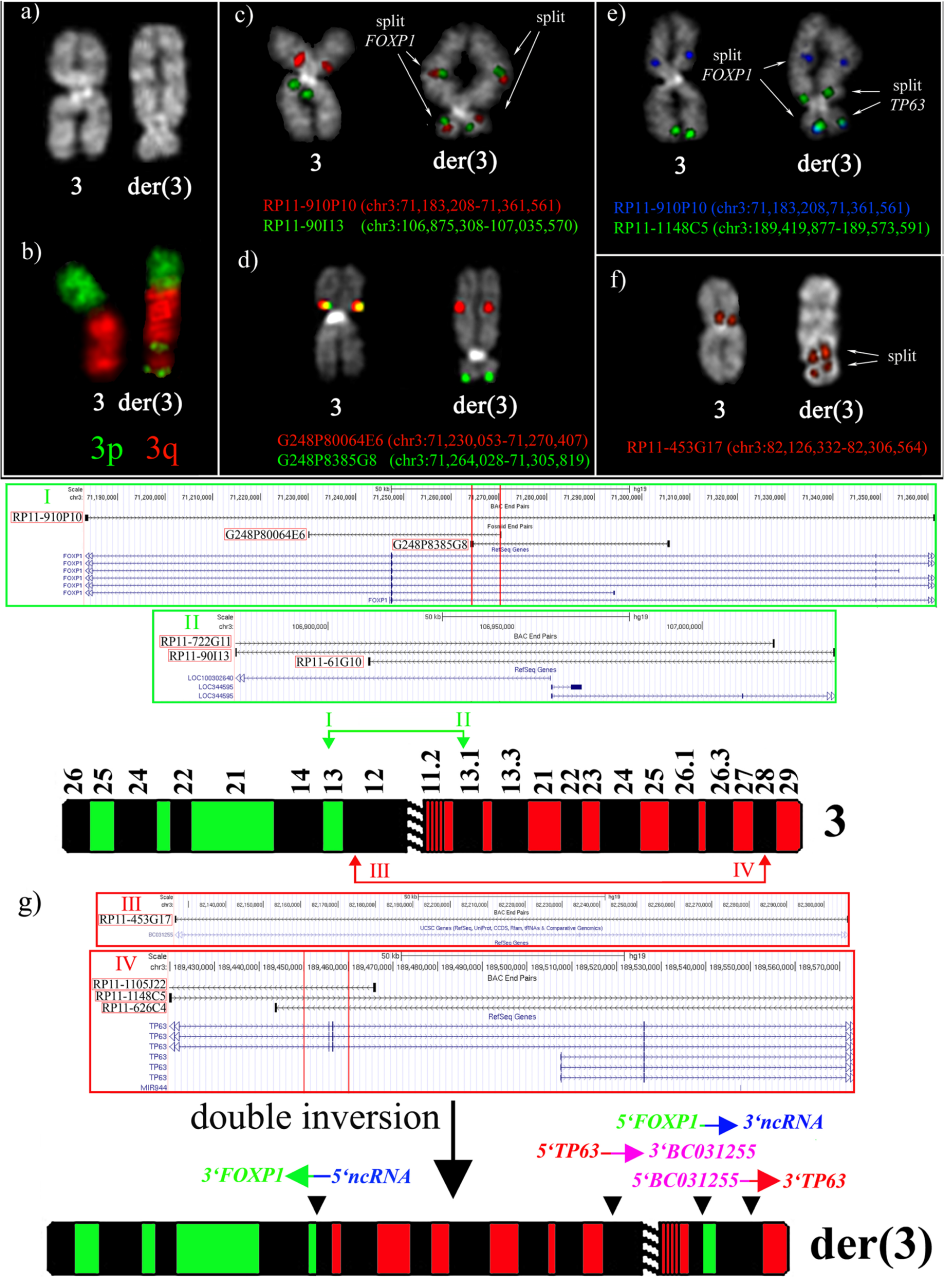
**Partial metaphases showing the normal and the rearranged chromosome 3. (a)** Q-banding; **(b)** FISH cohybridization experiment, performed as previously described [5], using commercial PCP probes specific for 3p (Kreatech, prod. No. KBI-30104, green), and 3q (Kreatech, prod. No. KBI-30105, red); **(c, d)** FISH experiments with BAC (c) and fosmid (d) clones to define the breakpoint regions in chromosome bands 3p13 and 3q13.12; **(e, f)** FISH cohybridization experiments with probes defining the breakpoints in bands 3p12.2 (e) and 3q28 (f); **(g)** Schematic representation of the double inversion leading to the formation of the der(3) chromosome. I, II, III, and IV refer to contig maps of BAC and fosmid clones of the breakpoint regions in chromosome bands 3p13, 3q13.12, 3p12.2, and 3q28, respectively. The clones used in FISH are indicated by red rectangles; the red arrows point on the intervals (defined by the red vertical lines) containing the breakpoints.

Here we describe a patient with an aggressive form of MDS. The BM karyotype, in addition to 5q deletion, showed an acquired abnormal chromosome 3 (Figure 1a). This abnormality led to the concurrent alteration of the *FOXP1* and *TP63* genes, never before reported in the literature.

## Case presentation

The patient, a 79-year woman, was admitted to our hospital in January 2010. Her blood count showed: WBC 10 × 10^9^/l with 1% myeloblasts, Hb 8.5 g per 100 ml, and platelets 135×109/l. Histological sections indicated that the BM was cellular at 80%, including numerous dystrophic megakaryocytes, reduction of normoblastic erythroid elements, and increase of myeloid immature cells with dysplastic morphology. Moreover, a diffuse and severe reticulin fibrosis was observed, and a diagnosis of myelodysplasia with myelofibrosis was made. The BM aspirate at MDS diagnosis had the karyotype: 46,XX,inv(3)(p?26q?13),del(5)(q31q35)[20].ish del(5)(q31.2q31.2)(EGR1-). The complex chromosome 3 rearrangement was found in all the metaphases, strongly suggesting its role as a driver mutation. Notably, this patient showed neither deletions nor point mutation at *TP53* (exons 4–8), known to be associated with an increased risk of leukemic evolution in MDS with del(5q) [1]. However, the patient, treated with hydroxyurea, progressed quickly towards AML in December 2011, with the following blood count: WBC 77 × 10^9^/l with 21% myeloblasts, Hb 8.1 g per 100 ml, and platelets 19x109/l. The karyotype at this stage was not available, due to the lack of metaphases in the BM. Immediately after, the patient died for a fatal cerebral hemorrhage.

CN analysis on the BM genomic DNA of the patient was accomplished on a Genome-wide human SNP array 6.0 according to manufacturer protocols (Affymetrix, Santa Clara, CA, USA). The resulting data, analyzed by the Genotyping console software V.4.1.3.840, and by Chromosome Analysis Suite V. CytoB-N1.2.0.225 using the GRCh37/hg19 genome sequence, confirmed the occurrence of a 5q23.1-q33.3 deletion. Additionally, CN variations within *JAK2* and *KMT2A* (also known as *MLL*) (CN state 1 and 3, respectively) were observed. FISH, performed as previously described [5], excluded the involvement of *JAK2* and *KMT2A* in further rearrangements leading, for instance, to fusion genes (data not shown), but it could not confirm the CN variation (18.8 and 32.6 Kb, respectively). We conclude that, apart from the deletion of chromosome 5, the complex rearrangement of chromosome 3 was the only relevant structural rearrangement in the bone marrow cells of the patient.

Very few CN switches were observed either on chromosome 3 or the rest of the genome. Due to the poor quality of the DNA, massive whole genome sequencing was not performed. However, the genesis of this rearrangement was not due to chromothripsis, because we observed less than ten CN variations [6] along the entire chromosome. The lack of mutation within *TP53*, previously reported as strongly associated with chromothripsis in AML [6], further reinforced this conclusion.

FISH analysis with chromosome paints for 3p and 3q revealed a complex reorganization of chromosome 3 (Figure 1b). Reiterative FISH assays with BAC (Roswell Park Cancer Institute [RPCI]-11 Human Male Bac Library, Buffalo, NY) and fosmid (WIBR2 Human Fosmid Library) probes, chosen according to the GRCh37/hg19 sequence and obtained from the BACPAC Resource Center (http://bacpac.chori.org), allowed us to map four breakpoint regions.

The first region was mapped within the overlap between RP11-910P10, G248P80064E6 and G248P8385G8 (Figures 1c, d, g), upstream to the coding sequence of the *FOXP1* gene (Figure 1g). *FOXP1* encodes for a transcription factor playing important roles in the regulation of tissue-specific gene transcription during cell growth and differentiation [7].

The second breakpoint region was located within the overlapping clones RP11-90I13 (Figure 1c), RP11-722G11, and RP11-61G10, encompassing the 5′ portions of the non coding RNA *LOC100302640* and *LOC344595* genes, with opposite transcriptional orientation, and with unknown function (Figure 1g).

We conclude that an initial inversion event [inv(3)(p13q13.12)] led to the juxtaposition of the *FOXP1* coding region to the 5′ portion of a non coding RNA gene.

The third and forth breakpoints were mapped, respectively, within the overlapping region between RP11-1148C5, RP11-1105 J22, and RP11-626C4 (Figures 1e,g). They encompassed the *TP63* gene (Figure 1g), and RP11-453G17 (Figure 1f,g), covering the first intron of the non-coding RNA *BC031255* gene, with unknown function. *TP63* is a member of the *TP53* family of transcription factor genes, encoding by alternative promoters for two main isoforms, *ΔNp63* (*p40*) and *TAp63*. The TAp63 proteins transactivate the majority of the TP53 target promoters inducing cell cycle arrest and apoptosis. On the contrary, the ΔNp63 isoforms seem to counteract the transactivation activities of TP53 and TAp63 proteins, possibly conferring a proliferative advantage on cancer cells [8]. Notably, the breakpoint within *TP63* was mapped upstream to the *ΔNp63* transcription start site (Figure 1g)*.*

The second inversion event [inv(3)(p12.2q28)] led to the rearrangement of *TP63* and *BC031255,* with the same transcriptional orientation.

Interphase FISH with the RP11-910P10 clone also showed the splitting of the probe in 94% and 74% of cells, respectively in the BM and in the peripheral blood (PB) at diagnosis (data not shown). This result disclosed that the abnormal cell clone was already present in the PB at onset, confirming the aggressiveness of the MDS disease.

**Figure 2:**
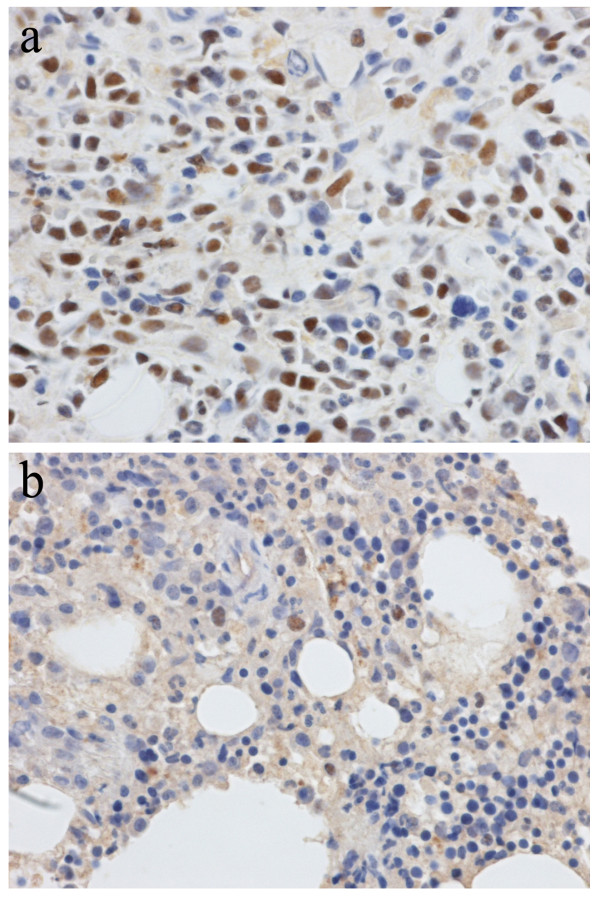
**Pictures of immunohistochemistry assays (600× magnification) performed with anti-TP63 (all isoforms) antibody on the bone marrow biopsie. (a)** the patient under study (no.8447); **(b)** the MDS case no. 7737 with del(5q) and trisomy 21 (see Additional file 1).

**Figure 3:**
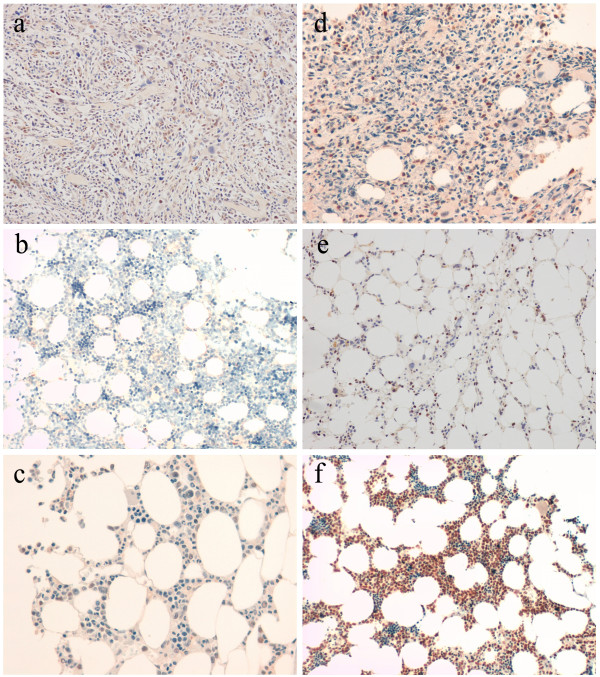
**Pictures of immunohistochemistry assays (200× magnification) performed with anti-FOXP1 antibody showing different levels of protein expression in the bone marrow biopsies of investigated cases. (a)** patient under study (level 3); **(b)** normal BM (level 0); **(c)** MDS case no. 558/10 with del(5q) as a sole cytogenetic abnormality (level 1); **(d)** MDS case no. 7737 with del(5q) and trisomy 21 (level 2); **(e)** MDS case no. 2374 with del(5q) and monosomy 7 (level 3); **(f)** AML case no. 635/12 with normal karyotype (level 4). (see Additional file 1).

The lack of RNA material precluded the possibility of investigating genes expression patterns. However, we evaluated *FOXP1* and *TP63* expression by immunohistochemistry analysis (Figures 2 and 3). The patient was negative for the expression of all ΔN isoforms of TP63 (with A00112 anti-p40 rabbit polyclonal antibody, Scytek, UT, USA) (data not shown). However, the patient was positive to the common region to all TP63 isoforms (with NCL-p63 anti-p63 mouse monoclonal antibody, Novocastra, Milan) (Figure 2a), as well as for FOXP1 (with E19062 rabbit anti-FOXP1 polyclonal antibody, Spring Bioscience Corp., CA, USA) (Figure 3a). For FOXP1, we found a prevalent positivity of myeloid precursor cells, present in a high percentage in the bone marrow of the patient.

We performed the same analysis on four normal BM, as well as in additional MDS/AML cases with normal karyotypes, or with 5q- as a sole cytogenetic abnormality, or with additional changes (Additional file 1).

The overall results showed that TP63 was negatively expressed in all the control cases (Figure 2b and Additional file 1). Conversely, FOXP1 was negatively expressed only in normal BM (Figure 3b) and one MDS case with a normal karyotype (Additional file 1). Of note, the remaining cases showed a variable level of protein expression (from 1 to 4, Additional file 1), directly proportional to the percentage of myeloid precursor cells found in each case [level 1 (10%); level 2 (10-50%); level 3 (50-80%); level 4 (more than 80%). A low positivity level was occasionally found in megacaryoblasts, megacaryocytes, and in a few cases in rare erytroid precursors.

Our overall results showed that AML cases in our cohort displayed higher expression levels (from 2 to 4) of FOXP1 than MDS (from 0 to 3). In particular, our patient showed the highest FOXP1 expression level among MDS cases (level 3), i.e. she had a high percentage of positive myeloid precursors in her bone marrow.

Moreover, we observed that the majority of the MDS/AML patients refractory to therapies, including our case, showed the highest FOXP1 expression levels [3–4 rather than 1–2, respectively in 6/11 (54%) versus 3/9 (33%)]. According to these preliminary results, which need to be confirmed by additional research, we hypothesize a possible involvement of this gene in the progression of myeloid diseases.

## Conclusions

To the best of our knowledge, we describe here the first MDS case with 5q- and a rearrangement of chromosome 3 involving *FOXP1* and *TP63.*

*FOXP1* was already described as fused to either the *PAX5* or the *ABL1* gene in B-ALL, and to the immunoglobulin heavy chain locus in lymphomas [7]. Strikingly, the breakpoint position in the present case was the same as in lymphomas accompanied by the gene upregulation. In myeloid malignancies, *FOXP1* was reported as a target of deletion in both AML [9] and in myeloproliferative neoplasms [10].We also report here for the first time that *FOXP1* is deregulated in MDS/AML cases. We presently have little information concerning the role of *TP63* isoforms in myeloid cells. In B-cells, upregulation of TAp63 isoforms increased survival by activating *BCL2* expression [11]. Mutations of *TP63* were described only in CML blast crisis [12].

In summary, we report here a notable MDS case, which rapidly evolved to AML, harboring 5q- and dysregulation of both *FOXP1* and *TP63*.

We hypothesize that there is a possible role of *FOXP1* and *TP63* upregulation in the disease progression in this patient, as well as a potential involvement of *FOXP1* in the evolution of MDS/AML and response to therapy, to be confirmed by additional experiments on a larger cohort of patients.

### Ethics statement

This study was performed in agreement with the Declaration of Helsinki, and approved by the Ethical Committee at the National Cancer Research Centre “Giovanni Paolo II”, Bari (Prot. No. 449/2013), and by the Ethical Committee at the “Seràgnoli” Institute of Hematology of Bologna (Prot. No. 253/2013/O).

Written informed consent was obtained from the patient for publication of this Case report and any accompanying images. A copy of the written consent is available for review by the Editor of this journal. Moreover, written informed consent was obtained for the control bone marrow samples and the additional MDS/AML cases.

## Abbreviations

(MDS): Myelodysplastic Syndromes; (5q)]: Deletion 5q [del; (BM): Bone marrow; (WBC): White blood cells; (Hb): Hemoglobin; (AML): Acute myeloid leukemia; (CN): Copy number; (FISH): Fluorescence in situ hybridization; (BAC): Bacterial Artificial Chromosome; *(FOXP1*): *forkhead box P1*; (*TP63*): *Tumor protein p63*; (PB): Peripheral blood; *(JAK2)*: *Janus kinase 2*; *(KMT2A)*: *Lysine (K)-specific methyltransferase 2*.

## Competing interests

The authors declare that they have no competing interests.

## Authors’ contributions

ALA performed FISH analysis, and analysed the data; CLC provided the sample material and conceived the study; EM and CA evaluated immunohistochemistry results; PI and AG critically revised the manuscript; CM, NT, GDT, and CM provided a substancial contribution to the acquisition of data for the study; CD performed immunohistochemistry analysis; MC performed TP53 mutation analysis; LAM reviewed the TP53 mutation data; SS performed SNP array CGH analysis; ET analysed SNP array CGH data and edited the manuscript; CTS reviewed all the data, and wrote the manuscript. All authors read and approved the final manuscript.

## Pre-publication history

The pre-publication history for this paper can be accessed here:

http://www.biomedcentral.com/1471-2407/14/396/prepub

## Supplementary Material

Additional file 1 **List of AML/MDS cases investigated for immunohistochemistry analysis.** The result for anti-FOXP1 antibody is reported in arabic numbers (from 0 to 4). Anti-TP40 and anti-TP63 antibodies result is expressed by + (positive) or – (negative). Click here for file

## References

[B1] Kulasekararaj AG Smith AE Mian SA Mohamedali AM Krishnamurthy P Lea NC Gaken J Pennaneach C Ireland R Czepulkowski B Pomplun S Marsh JC Mufti GJ TP53 mutations in myelodysplastic syndrome are strongly correlated with aberrations of chromosome 5, and correlate with adverse prognosis Br J Haematol 2013 160 5 660 672 10.1111/bjh.12203 23297687

[B2] Mallo M Cervera J Schanz J Such E Garcia-Manero G Luno E Steidl C Espinet B Vallespi T Germing U Blum S Ohyashiki K Grau J Pfeilstöcker M Hernández JM Noesslinger T Giagounidis A Aul C Calasanz MJ Martín ML Valent P Collado R Haferlach C Fonatsch C Lübbert M Stauder R Hildebrandt B Krieger O Pedro C Arenillas L Impact of adjunct cytogenetic abnormalities for prognostic stratification in patients with myelodysplastic syndrome and deletion 5q Leukemia 2011 25 1 110 120 10.1038/leu.2010.231 20882045

[B3] Mallo M Del Rey M Ibanez M Calasanz MJ Arenillas L Larrayoz MJ Pedro C Jerez A Maciejewski J Costa D Nomdedeu M Diez-Campelo M Lumbreras E González-Martínez T Marugán I Such E Cervera J Cigudosa JC Alvarez S Florensa L Hernández JM Solé F Response to lenalidomide in myelodysplastic syndromes with del(5q): influence of cytogenetics and mutations Br J Haematol 2013 162 1 74 86 10.1111/bjh.12354 23614682

[B4] Greenberg PL Tuechler H Schanz J Sanz G Garcia-Manero G Sole F Bennett JM Bowen D Fenaux P Dreyfus F Kantarjian H Kuendgen A Levis A Malcovati L Cazzola M Cermak J Fonatsch C Le Beau MM Slovak ML Krieger O Luebbert M Maciejewski J Magalhaes SM Miyazaki Y Pfeilstöcker M Sekeres M Sperr WR Stauder R Tauro S Valent P Revised international prognostic scoring system for myelodysplastic syndromes Blood 2012 120 12 2454 2465 10.1182/blood-2012-03-420489 22740453 PMC4425443

[B5] Storlazzi CT Albano F Dencic-Fekete M Djordjevic V Rocchi M Late-appearing pseudocentric fission event during chronic myeloid leukemia progression Cancer Genet Cytogenet 2007 174 1 61 67 10.1016/j.cancergencyto.2006.11.009 17350469

[B6] Rausch T Jones DT Zapatka M Stutz AM Zichner T Weischenfeldt J Jager N Remke M Shih D Northcott PA Pfaff E Tica J Wang Q Massimi L Witt H Bender S Pleier S Cin H Hawkins C Beck C von Deimling A Hans V Brors B Eils R Scheurlen W Blake J Benes V Kulozik AE Witt O Martin D Genome sequencing of pediatric medulloblastoma links catastrophic DNA rearrangements with TP53 mutations Cell 2012 148 1–2 59 71 22265402 10.1016/j.cell.2011.12.013 PMC3332216

[B7] Katoh M Igarashi M Fukuda H Nakagama H Cancer genetics and genomics of human FOX family genes Cancer Lett 2013 328 2 198 206 10.1016/j.canlet.2012.09.017 23022474

[B8] Candi E Dinsdale D Rufini A Salomoni P Knight RA Mueller M Krammer PH Melino G TAp63 and DeltaNp63 in cancer and epidermal development Cell Cycle 2007 6 3 274 285 10.4161/cc.6.3.3797 17264681

[B9] Bullinger L Kronke J Gaidzik V Dohner H Dohner K Comment on ‘Integrative genomic profiling of human prostate cancer’ Leukemia 2010 24 11 1970 1972 10.1038/leu.2010.194 20811405

[B10] Klampfl T Harutyunyan A Berg T Gisslinger B Schalling M Bagienski K Olcaydu D Passamonti F Rumi E Pietra D Jäger R Pieri L Guglielmelli P Iacobucci I Martinelli G Cazzola M Vannucchi AM Gisslinger H Kralovics R Genome integrity of myeloproliferative neoplasms in chronic phase and during disease progression Blood 2011 118 1 167 176 10.1182/blood-2011-01-331678 21531982

[B11] Shachar I Haran M The secret second life of an innocent chaperone: the story of CD74 and B cell/chronic lymphocytic leukemia cell survival Leuk Lymphoma 2011 52 8 1446 1454 10.3109/10428194.2011.565437 21417823

[B12] Yamaguchi H Inokuchi K Sakuma Y Dan K Mutation of the p51/p63 gene is associated with blastic crisis in chronic myelogenous leukemia Leukemia 2001 15 11 1729 1734 10.1038/sj.leu.2402265 11681414

